# Varicocele – a case for early intervention

**DOI:** 10.12688/f1000research.7179.1

**Published:** 2016-07-22

**Authors:** Phil V. Bach, Bobby B. Najari, Marc Goldstein

**Affiliations:** 1 Weill Cornell Medical College, New York, NY, 10065, USA

**Keywords:** Varicocele, hypogonadism, male infertility, microsurgery, varicocelectomy, testosterone, intracytoplasmic sperm injection, assisted reproductive technologies

## Abstract

Testicular varicocele, which is defined as the dilation of the veins draining the testicle, has long been associated with a detrimental effect on testicular function. Despite a lack of high-quality, prospective data, recent evidence has shed light on potential links between varicocele and male infertility and serum testosterone levels. Similarly, varicocele repair has increasingly been shown to have a beneficial impact on pregnancy rates, semen parameters, and on improving serum testosterone in adult men. Numerous studies have assessed the optimal technique for varicocele repair and the bulk of the evidence has shown the microsurgical inguinal/subinguinal approach to have the highest success rates, the lowest overall complication rates, and the lowest recurrence rates. The management of varicocele in adolescents remains a clinical conundrum, but contemporary evidence suggests early deleterious effects of varicocele on testicular function in some patients. Well-designed prospective trials are critical to delineate the true impact and role of varicocele repair on male infertility and hypogonadism in adult and adolescent men.

## Introduction

Testicular varicocele, which is defined as the dilation of the veins draining the testicle, occurs in approximately 15% of men in the general population ^1^. The prevalence of varicocele increases to 35% of men with primary infertility and between 75% and 81% of men with secondary infertility ^1^. Varicoceles have been associated with a progressive, detrimental effect on testicular function, affecting both spermatogenesis and Leydig cell function. However, despite being the most common known cause of male infertility, varicoceles and their pathophysiological effects on testicular function remain poorly understood, and controversy persists surrounding the appropriate management of varicocele.

In this review, we will examine the most recent literature surrounding varicoceles and their effects on various domains of testicular function, namely fertility and testosterone production. We will also explore recent evidence on the preferred technique for varicocele repair and the timing of varicocele repair in adolescents.

## Varicocele and fertility

Despite being the most common known cause of male infertility, 80% of men with varicoceles are fertile, a finding that complicates the true impact of varicocele on male fertility ^2^. A number of studies, including several randomized controlled trials (RCTs), have assessed the impact of varicocele repair on male fertility. While none of the studies have definitively determined the impact of varicocele repair on live birth rates, multiple studies have used pregnancy rates and semen parameters as surrogate outcomes for fertility.

10 RCTs comprising 894 men were included in a Cochrane Library meta-analysis that showed an increased odds ratio (OR) of 1.47 (95% confidence interval [CI] 1.05–2.05) of pregnancy following varicocele repair in men with subfertility, with a number needed to treat of 17 to achieve one pregnancy ^3^. However, their analysis was beset by low-quality evidence and significant heterogeneity among the 10 RCTs. For example, three RCTs included men with subclinical varicoceles only, while another two RCTs included men with normal semen parameters. Many RCTs were also plagued by small numbers, high dropout rates, and differing techniques of varicocele repair. A subgroup analysis performed by the same group of five RCTs restricted to 505 men with clinical varicoceles, abnormal semen parameters, and pregnancy rate as the primary outcome significantly favored varicocele treatment. In this subgroup analysis, the OR for achieving pregnancy increased to 2.39 (95% CI 1.13–3.38) with a number needed to treat of seven to achieve one pregnancy ^3^. Overall, the authors concluded that while their analysis appeared to support the practice of varicocele treatment in the management of male subfertility, the significant heterogeneity and low quality of the evidence available prevented them from definitively drawing conclusions.

Of note, the results of the latest Cochrane subgroup analysis mirror a 2007 meta-analysis that included two RCTs and three observational studies of infertile men with clinical varicoceles, abnormal semen parameters, and spontaneous pregnancy as the primary outcome. In this 2007 meta-analysis, the authors included high-quality observational studies in order to counteract the lack of reliable, high-quality RCT data and adhered to the Potsdam criteria to appraise the evidence and to reduce bias. They also found a significant advantage to surgical varicocele repair, with an OR for spontaneous pregnancy of 2.87 (95% CI 1.33–6.20, p=0.006) and a number needed to treat of 5.7 ^4^; 33% of patients who underwent surgical varicocele repair were able to conceive spontaneously compared to 15.5% of those who did not undergo surgical varicocele repair ^4^.

The impact of varicocele on semen parameters in infertile men has been studied extensively in both prospective studies and RCTs. Unlike the evidence assessing the impact of varicocele repair on clinical pregnancy rates, the evidence linking varicocele repair and improved semen parameters has consistently favored varicocele repair. A recent meta-analysis looking at semen parameters after varicocelectomy for infertile men found statistically significant improvements in sperm concentration (12.32 million sperm per milliliter [95% CI 9.45–15.19, p<0.0001]), total motility (10.86% [95% CI 7.07–14.65, p<0.0001]), and progressive motility (9.69% [95% CI 4.86–14.52, p<0.0001]) following repair of clinical varicocele ^5^. While the authors noted significant heterogeneity amongst the 22 prospective studies that looked at sperm concentration, 17 prospective studies examining total sperm motility, and five prospective studies examining progressive sperm motility, almost all of the studies assessed showed either a significant improvement or a trend towards improvement in the semen parameter studied following repair of a clinical varicocele. The meta-analysis also found that varicocele repair improves sperm parameters at a molecular level, with reductions in seminal oxidative stress and DNA damage as well as improvements in sperm ultramorphology ^5^.

The improvement in semen parameters also holds true in men undergoing varicocele repair for reasons other than infertility, such as testicular pain, discomfort, or scrotal mass. In a study of 268 men undergoing varicocele repair for an indication other than infertility, 63% had at least one abnormal parameter on pre-operative semen analysis and 76% showed improvement in at least one semen parameter after varicocele repair ^6^. The degree of impairment in sperm parameters has been repeatedly shown to be associated with varicocele grade, with repair of higher-grade (grade II–III) varicoceles resulting in more dramatic improvements in semen parameters ^7– 9^. Following varicocele repair, the most significant improvement in semen parameters occurs at 3 months, with little further improvement noted thereafter ^10^. While there appears to be a clear improvement in sperm concentration and motility, the measurement of semen parameters represents a surrogate measure of fertility, and the threshold of what degree of improvement results in improved pregnancy or live birth rates has yet to be established. Furthermore, the variability that exists between semen analyses may cloud the ability to draw robust conclusions from the meta-analysis.

Men with non-obstructive azoospermia (NOA) represent a unique subset of infertile men, in which assisted reproductive technologies (ARTs) such as *in vitro* fertilization (IVF) or intracytoplasmic sperm injection (ICSI) may be needed. However, in those men with NOA and a clinical varicocele, varicocele repair may result in the return of sperm to the ejaculate and preclude the need for a testicular retrieval of sperm prior to ART. A 2010 review of 11 retrospective, observational studies and 233 patients with NOA and clinical varicocele undergoing varicocele repair found that 39% of patients had a return of motile sperm to the ejaculate following varicocele repair and 6% of couples subsequently reported spontaneous pregnancy ^11^. Success, which was defined as a return of motile sperm to the ejaculate, was strongly associated with histopathological pattern within the testis, with late maturation arrest (46%) and hypospermatogenesis (55%) having significantly higher success rates than Sertoli cell only (11%) and early maturation arrest (0%) ^11^. A subsequent prospective study in 2012 by Abdel-Meguid also concluded that testicular histology was the only significant factor correlated with successful return of motile sperm to the ejaculate following varicocele repair in men with NOA and clinical varicocele ^12^. Abdel-Meguid was able to recover sperm in 54% of patients with hypospermatogenesis and 50% of patients with late maturation arrest, but was unable to recover sperm in patients with either Sertoli cell only or early maturation arrest ^12^. Unfortunately, while up to 40% of patients will have return of sperm to the ejaculate following varicocele repair, less than 10% of patients will have adequate sperm in the ejaculate and be able to avoid testicular sperm extraction (TESE) prior to ART ^13^.

Overall, despite the latest evidence being fraught with heterogeneity, there does appear to be a benefit to varicocele repair in the management of infertile men with abnormal semen parameters, with improvements seen not only in semen parameters but also in spontaneous pregnancy rates following varicocele repair.

## Varicocele repair in the setting of ART

With the increased use of ART in infertile couples, the role of varicocele repair in the setting of ART remains undefined. While varicocele repair in men with NOA appears to sometimes enable the return of motile sperm to the ejaculate, the impact of varicocele repair on sperm retrieval at TESE remains unclear. Schlegel *et al.* found no difference in sperm retrieval rates at TESE between men with and without prior varicocelectomy (60% in both groups) ^13^. In contrast, both Haydardedeoglu *et al.* (61% vs. 38%, p=0.01) and Inci *et al.* (53% vs. 30%, p=0.036) noted significantly higher rates of sperm retrieval at TESE in patients following varicocele repair ^14, 15^. Albeit small, a 2013 prospective study also noted a significantly higher rate of sperm retrieval at TESE in patients undergoing varicocele repair 3 months prior to TESE (58% vs. 27%, p<0.05) ^16^.

Whether or not varicocele repair improves pregnancy rates in couples using ART also remains controversial. Two large retrospective reviews have attempted to assess the impact of varicocelectomy on pregnancy and miscarriage rates during ICSI and have conflicting results. Pasqualotto *et al.* assessed 248 couples undergoing ICSI, of which 169 men had undergone varicocele repair and 79 men had clinical varicocele and found no differences between the two groups in terms of semen parameters (sperm concentration, motility, and morphology), pregnancy rates, implantation rates, or miscarriage rates ^17^. On the other hand, Esteves *et al.* investigated 242 couples undergoing ICSI, of which 80 men had undergone varicocele repair and 162 men had clinical varicocele, and noted significant improvements in the number of motile sperm (15.4 × 10 ^6^ vs. 6.7 × 10 ^6^, p<0.01), clinical pregnancy rate (60% vs. 45%, p=0.04), and live birth rate (46% vs. 31%, p=0.03). They also noted a decreased chance of miscarriage (OR 0.433, 95% CI 0.22–0.84) after varicocele repair ^18^. Both Haydardedeoglu *et al.* and Inci *et al.* also reported higher pregnancy rates with ICSI in men following varicocele repair ^14, 15^.

A small study of 58 couples undergoing intrauterine insemination also found a significant improvement in pregnancy rates (12% vs. 6%, p=0.04) and live birth rates (12% vs. 2%, p=0.007) for men who had varicocele repair when compared to those without varicocele treatment despite no differences in post-wash sperm counts ^19^. The authors suggested that the increased pregnancy and live birth rates could be secondary to improvements in sperm characteristics not measured on routine semen analysis.

The suggestion made by the above authors is in keeping with recent literature linking levels of sperm DNA damage to pregnancy rates. Evidence from meta-analyses has shown high levels of sperm DNA fragmentation to have a detrimental effect on the outcomes of IVF/ICSI, with decreased pregnancy rates and increased miscarriage rates ^20, 21^. While the pathophysiological mechanism linking varicocele to sperm DNA damage has yet to be clearly elucidated, increased reactive oxygen species (ROS)—resulting in increased seminal oxidative stress—are thought to play a central role. Varicocele is associated with higher levels of seminal ROS and sperm DNA damage, while repair of varicocele results in significantly decreased sperm DNA fragmentation ^22^. In a prospective study of 49 couples, Smit *et al.* showed a decrease in DNA fragmentation index from 35.2% to 30.2% (p=0.019) after varicocelectomy, with 37% of couples able to subsequently achieve spontaneous pregnancy and another 24% of couples able to achieve pregnancy with ART. The mean DNA fragmentation index was significantly higher in couples unable to achieve pregnancy either spontaneously or with ART ^23^. Kadioglu *et al.* also showed a significant decrease in DNA fragmentation index in 92 consecutive patients undergoing varicocele repair ^24^. With contemporary evidence suggesting that testicular spermatozoa have significantly less sperm DNA damage than ejaculated sperm and may result in higher pregnancy and live birth rates during ICSI, the insistence on using ejaculated sperm for ART has diminished ^25– 27^.

In summary, despite a lack of high-quality prospective RCT data, the literature does suggest a beneficial role for varicocele repair in decreasing sperm DNA fragmentation. While improved sperm DNA fragmentation could lead to improved pregnancy and live birth rates for couples pursuing ART, the data have not yet been able to demonstrate clearly defined associations between varicocele repair and reproductive outcomes in ART.

## Varicocele and testosterone

Apart from spermatogenesis, the other main domain of testicular function is the production of testosterone by the Leydig cells. While initial studies failed to definitively identify a correlation between serum testosterone levels and varicocele, a large World Health Organization study of 9034 men with and without varicocele presenting for male infertility evaluation suggested a progressive decline in Leydig cell secretion in men with varicocele that was not seen in those without varicocele ^28^. More recent studies have echoed the association between varicocele and low serum testosterone, with Tanrikut *et al.* finding a lower serum testosterone level in men with varicocele than in those without varicocele (416 ng/dL vs. 469 ng/dL, p<0.001) ^29^.

The association between varicocele and serum testosterone has also been repeatedly reinforced in recent retrospective and prospective studies looking at changes in serum testosterone levels after varicocele repair ( Table 1). The majority of these studies have shown significant increases in serum testosterone after varicocele repair, and a meta-analysis of nine studies and 814 patients found a statistically significant increase in serum testosterone of 97.48 ng/dL (95% CI 43.73–151.22, *p*=0.0004) after varicocele repair ^30^. The impact of varicocele repair on serum testosterone in adult men appears to be independent of age, with similar increases in serum testosterone following varicocele repair seen in men aged 16–65 with a baseline testosterone less than 400 mg/dL ^31^. While the change in serum testosterone following varicocele repair appears robust, many of the studies looking at this question are retrospective, with small patient numbers. For instance, of the nine studies in the aforementioned meta-analysis, eight were retrospective observational studies and seven included fewer than 80 patients ^30^. Furthermore, many of the studies include men with normal baseline serum testosterone levels and do not comment on hypogonadal symptoms, which makes it difficult to extrapolate the results to men with hypogonadism.

**Table 1.** Summary of the reported literature on varicocele repair and changes in serum testosterone.

Author, year	Age (years)	Number of patients	Baseline testosterone (ng/dL)	Post-operative testosterone (ng/dL)	Significance
Su *et al.* (1995) ^34^	35	53	319	409	p<0.001
Cayan *et al.* (1999) ^55^	29.5	78	563	837	p<0.01
Pierik *et al.* (2001) ^56^	35.5	30	542	571	NS
Fujisawa *et al.* (2001) ^57^	32	52	460	470	NS
Gat *et al.* (2004) ^32^	35.1	83	348	496	p<0.001
Lee *et al.* (2007) ^58^	39.6	18	360	416	NS
Di Besceglie *et al.* (2007) ^59^	28	38	650	660	NS
Ozden *et al.* (2008) ^60^	24	30	660	720	NS
Rodriquez *et al.* (2009) ^61^	23.5	202	648	709	NS
Hsiao *et al.* (2011) ^31^	35.6	106	309	431	p<0.001
Tanrikut *et al.* (2011) ^29^	36	325	358	454	p<0.001
Sathya *et al.* (2011) ^62^	30	100	177	301	p<0.001
Zohdy *et al.* (2011) ^63^	33.8	103	379	450	p<0.001
Hsiao *et al.* (2013) ^33^	36.4	59	308	418	p<0.001
Abdel-Meguid *et al.* (2014) ^64^	31.7	66	347	399	p=0.004

Abbreviations: NS, non-significant

As is evident in Table 1, the response in serum testosterone following varicocele repair varies dramatically. While patient age, laterality, and varicocele grade do not appear to affect the degree of increase in serum testosterone following varicocele repair, other factors such as baseline testosterone and testicular firmness are related to the degree of postoperative testosterone response ^29, 32, 33^. Hypogonadal men have a greater, statistically significant improvement in serum testosterone following varicocele repair when compared to eugonadal men ^31, 34^. Despite having similar preoperative serum testosterone, men with at least one firm testis, which likely represents at least one healthy testis, have also demonstrated a greater improvement in serum testosterone following varicocele repair compared to those with bilateral soft testes, which may represent end-stage testicular dysfunction (144 ng/dL vs. 10 ng/dL, p<0.005) ^34^.

The link between varicocele and serum testosterone levels suggested by the clinical studies cited above is corroborated by histological and animal studies. Testicular biopsies from patients with idiopathic varicoceles demonstrate a decreased tubular diameter, hyperplasia in the number of Leydig cells with cytoplasmic vacuolization and atrophy, and a decrease in the number of testosterone-positive Leydig cells ^35^. In Sprague-Dawley rats with an induced varicocele, intratesticular testosterone concentrations were significantly lower than in control rats, further suggesting an association between varicocele and impaired Leydig cell function ^36, 37^.

Taken together, despite the lack of definitive, high-quality data linking varicocele and hypogonadism, the evidence suggests that varicocele repair may increase serum testosterone levels in adult men across all age groups. Further prospective studies are needed to ultimately determine whether or not varicocele repair should be considered a potential treatment option for hypogonadal men.

## Varicocele repair techniques

Numerous options are available to the patient who has decided to undergo varicocele repair, including open retroperitoneal, open inguinal, laparoscopic retroperitoneal, microsurgical inguinal, microsurgical subinguinal, radiographic embolization, and sclerotherapy. The various techniques for varicocele repair have been exhaustively studied, though there remains a lack of large, high-quality prospective RCTs in this arena. A recent meta-analysis evaluated 35 studies (23 RCTs and 12 observational studies) that compared the various varicocele repair techniques ^38^. While the different techniques were not directly compared to one another in the meta-analysis, they were all compared to open retroperitoneal repair. Overall, the authors found that of the techniques for varicocele repair assessed, which included laparoscopic retroperitoneal and open conventional inguinal repair, radiographic embolization, and sclerotherapy, the microsurgical inguinal/subinguinal approaches had the lowest overall complication rates ^38^. The odds for varicocele recurrence were lowest for the microsurgical inguinal and subinguinal approaches when compared to the open retroperitoneal approach (OR 0.06, 95% CI 0.01–0.16 and OR 0.05, 95% CI 0.01–0.19, respectively), whereas the other repair techniques did not demonstrate significant differences in varicocele recurrence when compared to the open retroperitoneal approach ^38^. Similarly, the microsurgical inguinal and subinguinal approaches proved to be superior to open approaches for minimizing the risk of hydrocele formation. Two of the included studies in the meta-analysis reported no hydrocele formation after microsurgical inguinal varicocele repair, while microsurgical subinguinal repair dramatically outperformed open retroperitoneal repair (OR 0.05, 95% CI 0.00–0.42) ^38^. Tauber antegrade sclerotherapy was also very unlikely to be associated with hydrocele formation when compared to open retroperitoneal repair (OR 0.01, 95% CI 0.00–0.13) ^38^. While this meta-analysis encapsulates a comprehensive swath of the literature surrounding techniques for varicocele repair, it is limited by the quality of the studies included. Many of the RCTs are noted to be of poor quality, and there is heterogeneity amongst the studies in terms of patient selection and surgical technique, especially for the open retroperitoneal approach, where some studies did not use optical magnification or make efforts to spare the testicular artery. Ligation of the testicular artery, which can occur accidentally in 0.9% of microsurgical inguinal or subinguinal varicocele repairs, may result in testicular atrophy in 5% of patients ^39^.

Despite its limitations, the overall results of this recent meta-analysis are consistent with older studies that have also shown the microsurgical inguinal/subinguinal approach to have significantly higher pregnancy rates (42%), lower recurrence rates (1%), and lower hydrocele formation rates (0.4%) when compared to the other techniques for varicocele repair ^40^. The rationale behind the superior results with the microsurgical approaches is the improved visualization, which enables the ligation and division of all internal spermatic, external spermatic, cremasteric, and gubernacular veins while sparing the lymphatic structures. While the radiographic approaches (both embolization and sclerotherapy) can have promising initial results, our experience has seen many men recur later (typically after 2–5 years) with slow-filling veins that may be attributable to a failure to cannulate small collaterals and/or recanalization through the coils.

Since it was described in 2008 by Shu *et al.* ^41^, robot-assisted varicocele repair and its efficacy have been explored by various groups. While the small number of studies in the literature tout the elimination of hand tremor as a significant advantage to using the robotic platform in microsurgery, there remains a paucity of data on outcomes, complications, and comparisons to currently used techniques for varicocele repair ^41, 42^.

In spite of numerous limitations, the current data suggest that the microsurgical inguinal or subinguinal approach results in higher pregnancy rates and lower recurrence and complication rates, and should be the favored technique for varicocele repair. However, well-designed, large, prospective RCTs would be helpful to confirm these assertions.

## Varicocele repair in adolescents

With varicoceles being found in 17% of adolescent males ^43^ and with contemporary evidence suggesting links between varicocele and infertility in adult men, the question of when or whether to treat varicoceles in the adolescent population is critical. Currently, the accepted indication for varicocele repair in adolescents is objective evidence of diminished ipsilateral testicular size. The guidelines from the American Society for Reproductive Medicine advocate waiting for detection of changes in testicular size or semen analysis prior to recommending varicocele repair ^44^.

Disturbing data show that the deleterious effects of varicocele on testicular function are seen at an early age and may be progressive. Several groups have noted an increased prevalence of varicocele in men with secondary infertility when compared to those with primary infertility, suggesting a progressive, time-dependent decline in male fertility ^45, 46^. Further evidence of a progressive impact of varicocele on male fertility comes from a small longitudinal study that followed 13 men with normal semen parameters for 9–96 months. With time, the men demonstrated a statistically significant deterioration in sperm concentration and motility that went from being normal to abnormal ^47^. Differences in semen parameters such as motility, vitality, and morphology have been noted in men as young as 17–19 years of age with varicoceles ^48^. Two recent meta-analyses have also demonstrated abnormalities in semen parameters in adolescents with varicoceles, further reinforcing the concern regarding the early harmful effects of varicocele on male fertility ^49, 50^. As with adults, treatment of varicocele appears to improve semen parameters ^49^. Unfortunately, most studies in adolescents have short follow-up periods, making it difficult to ascertain with confidence whether the improvement in semen parameters is primarily due to the varicocele repair or whether the improvement would have happened without treatment as the adolescent testicle developed and matured over time.

While there is concerning evidence regarding both early and progressive detrimental effects of varicocele on male fertility, there is a lack of prospective, longitudinal data regarding the ultimate effect of adolescent varicocele on future adult fertility and pregnancy rates. Bogaert *et al.* studied the paternity status in a cohort of men who had been previously diagnosed with varicocele as adolescents and found no difference between survey respondents who had elected to undergo varicocele repair and those who were managed conservatively (78% vs. 85%, p>0.05) ^51^. Their findings suggest no role for varicocele repair in adolescents, and also support older data that show over 80% of those with varicocele remain fertile.

The current emphasis on differential testicular volume in the management of adolescent varicocele is also controversial. Recovery of testicular volume and resolution of differential testicular volume after varicocele repair is thought to be a sign of treatment success and a surrogate for restoration of normal testicular function. Support for this paradigm comes from a retrospective case-control study in which the testicular volumes and semen parameters were compared between 32 men who had previously undergone varicocele repair as adolescents, 26 men who had been diagnosed with varicocele as adolescents and observed, and 27 age-matched controls. Of the 32 men who underwent varicocele repair, 75% had ipsilateral testicular hypotrophy at the time of surgery. At a mean of 14.6 years after treatment, treated patients had no difference in testicular volume bilaterally, and had no differences in testicular volume or sperm concentration when compared to the control group. In contrast, the untreated group had persistent ipsilateral testicular hypotrophy and had significantly lower testicular volume and sperm concentration when compared to the treated and control groups ^52^. Conversely, Kolon *et al.* followed a group of 71 boys with varicocele and ipsilateral testicular hypotrophy with serial ultrasound for 2 years. While only 54% of boys initially had a discrepancy in testicular volume of under 15% initially, 85% of boys had testicular volume differentials of under 15% after 2 years, indicating that adolescent males have asynchronous testicular growth that often equalizes with time despite the presence of a varicocele ^53^. More recently, Kurtz *et al.* retrospectively analyzed the ultrasound-derived testicular volumes and semen parameters of 100 adolescents with clinically detectable, but untreated, left varicoceles. They noted a significant difference between the volumes of the right and left testicles (15.9±6.9 cc versus 14.4±6.9 cc, p<0.0001 for right and left testicle, respectively), with 31% of patients having a total volume differential greater than 20% ^54^. There were significant associations between both testicular volume differential greater than 20% (OR 2.1, 95% CI 1.02–4.12, p=0.04) and total testicular volume under 30 cc (OR 4.2, 95% CI 1.8–9.7, p<0.001) on the odds of having a total motile sperm count below 20 million/cc ^54^. The above studies suggest that while clinical varicocele in the adolescent population may impact testicular volume and function in some patients, significant gaps in our knowledge and ability to predict which adolescents with clinical varicocele are harmed and would most benefit from varicocele treatment remain.

The management of adolescent varicocele remains a challenging clinical conundrum, especially in light of the dearth of data regarding the long-term effects of varicocele on future fertility and hormonal function. However, concerning evidence suggesting potential early damage to testicular function behooves the urologic community to pursue prospective, longitudinal studies that will help elucidate when and in what population of adolescents varicocele repair would be most beneficial.

In our opinion, logic would dictate that with the high success rate and low morbidity of microsurgical varicocelectomy, the conservative treatment of large, grade III varicoceles in adolescents is, in fact, microsurgical repair. Undergoing microsurgical repair would ensure conservation of testicular function, and we consider the prevention of future infertility and androgen deficiency to be preferable to the treatment of testicular dysfunction after it occurs. Understandably, before a shift in the management paradigm of this magnitude can occur, high-quality prospective data assessing the reproductive and hormonal outcomes in cohorts of treated and untreated adolescents with clinical varicocele through to adulthood are needed to populate well-designed effectiveness models and analyses.

## Future directions

A recurring theme when examining the current evidence surrounding the impact of varicocele treatment on infertility and serum testosterone in adult and adolescent men is the poor quality and pervasive heterogeneity of the evidence, which precludes the derivation of robust conclusions from the available data. While the current flawed evidence suggests that varicocele repair may be beneficial for men with infertility or hypogonadism, we echo numerous authors in calling for well-designed, prospective clinical trials that can provide confirmatory answers to the longstanding questions surrounding the impact and role for varicocele repair in the management of men with infertility or hypogonadism.

At this point, the ideal RCT looking at the impact of varicocele repair in adult men would be multi-institutional and compare microsurgical, testicular artery-sparing varicocele repair to non-intervention, with semen analyses, serum testosterone testing, and hypogonadism symptom assessments done according to the validated criteria and methodologies established in the literature. To study the question of the impact of varicocele repair on infertility, the study population would include men with clinical varicocele and subfertility defined both clinically and by abnormal semen analysis, with female factors also being taken into account in the analysis. To study the question of the impact of varicocele repair on hypogonadism, the study population would include men with hypogonadism defined by both a low serum testosterone level and clinical symptoms of hypogonadism.

The impact of varicocele repair in adolescent men could be studied using a prospective cohort design. Adolescents with clinical varicocele at various centers that routinely either treat or do not treat varicocele would have their semen analyses, hormone levels, and reproductive outcomes followed prospectively through to adulthood using validated criteria and methodologies to assess semen analysis and serum testosterone.

## Conclusion

Despite the high prevalence of varicocele in the general population and years of study, many questions remain surrounding its management. Even with a lack of high-quality, prospective data, the mounting body of evidence suggests a growing role for microsurgical varicocele repair in men with infertility. More work is needed to continue building insight into the pathophysiology and effects of varicocele on both adult and adolescent men in order to determine the optimal treatment for patients with varicocele.

## Abbreviations

ART, assisted reproductive technology; CI, confidence interval; ICSI, intracytoplasmic sperm injection; IVF, *in vitro* fertilization; NOA, non-obstructive azoospermia; OR, odds ratio; RCT, randomized controlled trial; ROS, reactive oxygen species; TESE, testicular sperm extraction.
